# Propiconazole-Induced Testis Damage and MAPK-Mediated Apoptosis and Autophagy in Germ Cells

**DOI:** 10.3390/cells14201624

**Published:** 2025-10-17

**Authors:** Won-Young Lee, Ran Lee, Hyeon Woo Sim, Hyun-Jung Park

**Affiliations:** 1 Department of Livestock, Korea National University of Agriculture and Fisheries, Jeonbuk 54874, Republic of Korea; leewy81@korea.kr (W.-Y.L.); ranran2424@gmail.com (R.L.); 2 Department of Animal Biotechnology, College of Life Science, Sangji University, Wonju-si 26339, Republic of Korea; opensesim@gmail.com

**Keywords:** propiconazole, spermatogenesis, germ cell, apoptosis, autophagy

## Abstract

**Highlights:**

**What are the main findings?**

- PRO causes testicular toxicity and sperm dysfunction by triggering autophagy-mediated apoptosis in germ cells.
- This is the first report demonstrating PRO’s effects on sperm motility and autophagy in mouse spermatogonia.

**What are the implication of the main findings?**

- Exposure to propiconazole poses a potential risk to male reproductive health, mediated in part by autophagy-dependent toxicity.

**Abstract:**

Propiconazole (PRO), a triazole fungicide, controls fungal diseases by disrupting ergosterol production in fungal cells. It is used in crops such as cereals and fruits. However, there are concerns regarding its potential to disrupt the endocrine system and cause reproductive toxicity. This study examined the effects of PRO on mouse testes, germ cells, and GC-1 spermatogonia. After eight weeks, PRO reduced testicular diameter and downregulated key germ cell genes (Sall4, Piwil, Nanos2, and Dazl). A histological examination revealed smaller seminiferous tubules and fewer SALL4+ cells. PRO also impaired steroidogenesis by downregulating genes (StAR, Cyp11a1, 3β-HSD1) and reducing sperm motility, with a decline in Velocity Straight Line (VSL), Linearity (LIN), Straightness (STR), and motile sperm. PRO caused dose-dependent cytotoxicity in GC-1 spermatogonia, decreased proliferation, and increased apoptosis, marked by cleaved caspase-3 and BAX. PRO also induced autophagy, as presented by elevated levels of autophagy-related genes (LC3 and ATG12) and proteins (ATG5 and LC3A/B). 3-Methyladenine (3-MA), an autophagy inhibitor, downregulates levels of autophagy- and apoptosis-related proteins when 3-MA and PRO are simultaneously treated in vitro. This suggests that both apoptosis and autophagy contribute to PRO-induced testicular cytotoxicity. This study is the first to detail that PRO affects sperm motility in mice and induces autophagy-mediated apoptosis in GC-1 spg.

## 1. Introduction

Pesticides are widely utilized globally in agriculture for crop protection and public health for disease Ctrl. These pesticides encompass a variety of chemicals, primarily insecticides, such as organochlorines, organophosphates, pyrethroids, carbamates, and neonicotinoids; herbicides, such as acid herbicides; and fungicides, such as azoles. Propiconazole (PRO), a triazole compound, is widely used in agriculture as a systemic fungicide for various crops, including turfgrasses cultivated for seeds, esthetic purposes, and athletic fields, as well as in mushrooms, corn, wild rice, peanuts, almonds, sorghum, oats, pecans, apricots, peaches, plums, and prunes. PRO is particularly utilized in both agriculture and wood preservation; its annual usage is estimated to be around 860 tons in the United States [1,2].

PRO can have harmful effects on mammals, fish, insects, zooplankton, and mollusks [3]. According to the results of rodent experiments, the daily administration of PRO at 150 mg/kg caused hepatomegaly in both mice and rats, whereas a dose of 450 mg/kg led to hepatic necrosis in rats [4]. Furthermore, PRO exposed in HepG2 cells, a human hepatic cell line, increased oxidative stress and elevated the levels of inflammatory and fibrotic markers. It induced morphological changes through oxidative stress and TGF-β/Smad pathways, reducing E-cadherin and increasing vimentin and snail. In rats, PRO causes liver fibrosis, epithelial–mesenchymal transition, and collagen deposition. PRO exposure may, therefore, impair liver function in humans [5].

In *Xenopus tropicalis* models, PRO induced endocrine activity during a critical period of sexual development, resulting in lasting reproductive and hepatic effects observed two months post-exposure. These adverse outcomes are likely linked to the endocrine mode of action. This study highlights the risk of male reproductive toxicity in amphibians due to exposure of tadpoles to azole compounds, including PRO [6]. Furthermore, Karlsson et al. reported that the adult male offspring (F1) of fathers exposed to pesticides presented reduced body size, lower fertility, and signs of endocrine disruption. These effects extended to the grand offspring (F2), confirming transgenerational effects on amphibians. F2 males exhibited increased weight, higher fat body palmitoleic-to-palmitic acid ratio, and lower plasma glucose levels. This study provides significant cross-species evidence of paternal epigenetic inheritance and transgenerational toxicity due to pollutants, underscoring the complex role of environmental contamination in amphibian extinction [7].

There are very few reports on the concentrations of PRO in the human body. PRO has been detected in hair samples from the general population. Consumption of PRO was measured in the hair of women in certain regions of China and was found to be 0.36 pg/mg [8].

The reported effects of triazole exposure in rodents include reproductive toxicity for myclobutanil and triadimefon and the carcinogenicity of propiconazole and triadimefon. In the two-generation rat reproductive studies, myclobutanil exposure led to testicular and prostatic atrophy, reduced litter size, and decreased pup weight gain [9]. Two-generation reproductive studies in rats treated with triadimefon have revealed decreased fertility, smaller litter sizes, reduced neonate viability, and increased serum testosterone levels in the F1 generation [3]. Several studies have demonstrated that PRO affects various aspects of steroidogenesis and influences the activity of specific hormone receptors, indicating its potential as an endocrine disruptor. Furthermore, PRO antagonizes the androgen receptor [10,11], exhibits antiestrogenic properties, and inhibits estradiol (E2) and testosterone (T) synthesis [12,13]. Moreover, in vivo studies have indicated that PRO exposure regulates steroid hormone levels and affects the male reproductive organ weight in rats [9,14]. Exposure to PRO has previously been demonstrated to affect the weight and development of gonads in rats [15].

Costa et al. reported the reproductive toxicity of propiconazole in male rats. Rats were divided into three groups and received daily gavage treatments with corn oil (Ctrl group), propiconazole at 4 mg/kg (PRO 4), or propiconazole at 20 mg/kg (PRO 20) from postnatal days 50 to 120. Body weight gain, sexual behavior, plasma testosterone and estradiol levels, organ weight, sperm count and morphology, and testicular histomorphology were monitored. There was an increase in the abnormal tail morphology of sperm in the PRO 4 group, as well as the weight of the seminal vesicles and vas deferens, along with a decrease in estradiol levels. Sexual behavior was affected only in the PRO 20 group [16]. Although previous studies have reported the reproductive toxicity of PRO in male rats, the findings of the present study are limited.

This study elucidates the reproductive toxicity of PRO, a triazole fungicide widely used in agriculture. It systematically examined the effects of PRO on various testicular cell types, including germ cells, Sertoli cells, and Leydig cells. The findings provide the first evidence that PRO directly impairs sperm motility and triggers autophagy-mediated apoptosis in spermatogonial cells. These results highlight autophagy as a key mechanism underlying PRO-induced testicular dysfunction, offering new insight into pesticide-related male infertility.

## 2. Materials and Methods

### 2.1. Animals and Treatment

Seven-week-old C57/BL6 male mice were sourced from Daehan Biolink Co., Ltd. (Daejeon, Republic of Korea) and acclimatized for a week. The animal experiment involved five Ctrl animals and five experimental animals and was repeated three times. They were housed under a 12 h light:12 h dark cycle at a constant temperature of 21 ± 1 °C. All animal procedures were performed following the guidelines of the Institutional Animal Care and Use Committee of Sangji University (approval number # 2024-4). PRO (Sigma-Aldrich, St. Louis, MO, USA) was dissolved in a minimal volume of dimethyl sulfoxide (DMSO, 10%) and phosphate-buffered saline (PBS, 90%) to achieve the final concentrations. The mice were divided into two groups (each containing ten mice) and received daily oral injections over eight weeks—Control (Ctrl); phosphate-buffered saline (10% of DMSO and 90% of PBS) and Propiconazole (PRO); 150 mg/kg/Body weight of PRO. PRO dosages were determined based on previous studies [4,17]. Body weight was monitored weekly, and testis weight was measured at the end of the eight weeks following euthanasia. Serum was harvested to measure testosterone levels. Testis tissue was fixed in 4% formaldehyde or frozen at −80 °C for isolation of protein and RNA.

### 2.2. Sperm Collection and Motility Assessment

Sperm motility analysis was performed using 18 epididymal sperm collected from nine randomly assigned male mice (Ctrl and experimental groups). Sperm were collected from the cauda epididymis in M2 medium (Sigma, St. Louis, MO, USA) and incubated at 37 °C for 90 min to maintain optimal sperm viability and motility. Sperm motility and kinetic parameters were evaluated using a phase-contrast microscope (Nikon 80i, Tokyo, Japan) integrated with a computer-assisted sperm analysis (CASA) system. Sperm samples were diluted to a final concentration of 1 × 10^7^/mL, and 20 µL aliquots were collected and analyzed using CASA software (DITECT, Tokyo, Japan) sperm motility analysis software. For each sample, at least five randomly selected fields and at least 500 sperm trajectories were evaluated. Analysis was performed at a frame rate of 25 frames per second (Hz), with 25 frames acquired per field. The CASA software settings defined the cell size range as 5–200 µm^2^. Motility parameters included linear velocity (VSL), curvilinear velocity (VCL), mean path velocity (VAP), linearity (LIN = VSL/VCL), straightness (STR = VSL/VAP), and fluctuation (WOB = VAP/VCL), which reflects the variability of sperm trajectories relative to the mean path.

### 2.3. Cell Culture and Treatment

The mouse GC-1 spermatogonia (spg) cell line (passage No. 30) was obtained from the Korean Cell Line Bank (KCLB, Seoul, Republic of Korea) and cultured in Dulbecco’s modified Eagle’s medium supplemented with 10% fetal bovine serum and 1% penicillin-streptomycin. The cells were maintained in a humidified atmosphere of 5% CO_2_ at 37 °C. PRO was dissolved in DMSO to create a 100 mM stock solution, which was then diluted to the required concentration (100 μM, 1 μL/mL) with cell culture medium before use in cell culture for 24 h. 3-Methyladenine (3-MA), a chemical that inhibits autophagy, was used freshly added to the medium at a concentration of 1 mM. The Ctrl group cultured in medium containing 0.1% DMSO.

### 2.4. Histological Analysis and Immunostaining

Testis samples were fixed in 4% paraformaldehyde overnight at 4 °C, then gradually dehydrated, embedded in paraffin, and sectioned into 5 μm-thick slices using a Leica microtome (GMBH, Nussloch, Germany). The sections were stained with hematoxylin and eosin. Five distinct areas of the testicular tissue were examined under a microscope by an analyst blinded to experimental conditions. Immunostaining was performed following previously established protocols [18,19], and the stained sections were viewed under a light microscope (Olympus IX73, Tokyo, Japan). Quantitative analyses were performed on more than four samples from each experimental group. For each sample, testicular cross-sections with at least 40 tubules from four sections were evaluated for analysis. Detailed information on the primary and secondary antibodies is provided in Table 1.

GC-1 spg cells grown on chambered cover glasses were exposed to 100 μM of PRO. After 24 h, the cover glasses were washed with PBS and immediately fixed with 4% formaldehyde for 15 min at 24 °C. The cells were then washed and stained with the first antibody in a blocking buffer (PBS containing 0.05% Triton X-100 and 1% bovine serum albumin) for 24 h at 4 °C. After being washed with PBS, a secondary antibody was applied for 1 h at 24 °C. The samples were mounted with DPAI (Vector Laboratories Inc., Burlingame, CA, USA) and images were captured using a fluorescence microscope (Olympus IX73, Tokyo, Japan). Detailed information on the antibodies is presented in Table 1. Antibodies were purchased from Cell Signaling Technology (Danvers, MA, USA), Abcam (Cambridge, UK), and Santa Cruz Biotechnology (Dallas, TX, USA).

### 2.5. Western Blotting

Proteins intended for Western blot analysis were extracted using RPIA buffer (Thermo Fisher Scientific, Philadelphia, PA, USA) combined with a protease inhibitor cocktail (Roche, Mannheim, Germany). Each protein sample (30 μg) was loaded onto 10% or 4–20% acrylamide gels (Bio-Rad, Hercules, CA, USA; #456–1096) and subsequently transferred to PVDF membranes. The membrane was then incubated with a primary antibody for 18 h at 4 °C (refer to Table 1 for the specifics of the primary antibodies used). Following the primary antibody incubation, the membrane was treated with secondary antibodies for 1 h at 24 °C. The Western Pico chemiluminescent substrate (Thermo Scientific, Rockford, IL, USA) was used for the protein band detection, and images were captured using a Bright™ Imaging System (Thermo Fisher Scientific, Inc., Waltham, MA, USA). The anti-β Actin antibody was employed as a normalization Ctrl.

### 2.6. Quantitative PCR Analysis

RNA was extracted using a RNeasy Mini Kit (Qiagen, Hilden, Germany) with an on-column DNase treatment (Qiagen). Reverse transcription to produce complementary DNA (cDNA) was performed using a RevertAid First Strand cDNA Synthesis Kit (Thermo Scientific, Rockford, IL, USA). Quantitative real-time PCR (qPCR) was performed on a QuatStudio 1 instrument (Applied Biosystems, Foster City, CA, USA) using SYBR mixture (Thermo Fisher Scientific, Waltham, MA, USA). The reaction conditions and data analysis methods were outlined in our previous study [18]. Quantification was normalized to endogenous GAPDH levels, with data presented on a log2 scale. The primers used, including those designed using Primer3 (https://primer3.ut.ee/), are listed in Table 2.

### 2.7. Flow Cytometry

A flow cytometer (CytoFLEX; Beckman Coulter, Inc., Miami, FL, USA) was used for annexin V-fluorescein isothiocyanate (FITC) and propidium iodide (PI) staining to identify apoptotic cells. After 24 h of PRO exposure, cells were collected and rinsed with DPBS. After annexin V FITC and propidium iodide (PI) staining, the cells were analyzed using flow cytometry. The 3 × 10^4^ cells were analyzed in a single analysis. Compensation was performed using unstained Annexin V apoptotic and necrotic Ctrl. After performing a compensation reaction using unstained Annexin V apoptosis and necrosis Ctrl, the final gated cells were analyzed for cell viability, apoptosis, and necrosis. This study was performed three times per sample and repeated five times. The detailed procedure for Annexin V-FITC staining has been previously outlined by Park et al. [20].

### 2.8. Measurement of Testosterone

Blood samples were collected by cardiac puncture. The serum was separated from whole blood. Serum levels (0–1000 pg/mL) of testosterone were measured using a mouse testosterone enzyme-linked immunosorbent assay (ELISA) kit (Cusabio Biotech, Houston, TX, USA). To each well, 50 μL of standard or serum sample was added, followed by 50 μL of biotin conjugate, and incubated at 37 °C for 30 min. After washing three times, 100 μL of HRP-avidin was added, incubated for another 30 min at 37 °C, and then washed five more times. Next, 90 μL of TMB substrate was added and incubated in the dark for 20 min, and the reaction was terminated by adding 50 μL of stop solution. Absorbance was measured at 450 nm using a microplate reader (Epoch, Bio Teck, Winooski, VT, USA). Wavelength compensation was set to 570 nm and exclude the measured values.

### 2.9. Statistical Analysis

The results are expressed as mean ± standard error (SEM), derived from at least three independent experiments, each performed in triplicate. One-way analysis of variance (ANOVA) was used to compare means, and Tukey’s post hoc test was used for further analysis. Statistical tests were conducted using SPSS version 15.0 for Windows (IBM Corp, Somers, NY, USA). Statistical significance was denoted by * *p* < 0.05, ** *p* < 0.01, and *** *p* < 0.001.

## 3. Results

### 3.1. Effects of PRO on Mice Testis and Germ Cell

**Figure 1:**
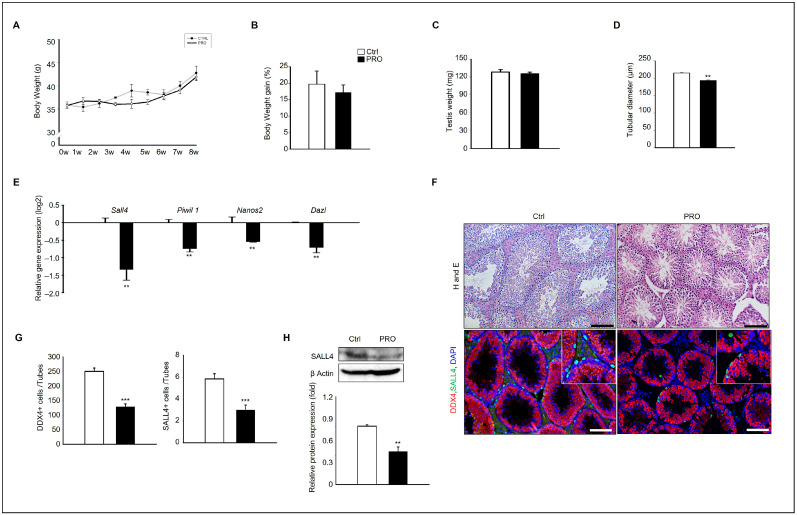
PRO exposure affects the testicular germ cell. (**A**) Body weight and (**B**) Body weight gain from each experimental group. Data are presented as mean ± SD. (**C**) Testis weight and (**D**) Seminiferous tubular diameter were measured from each group. Data are shown as mean ± SD. ** *p* < 0.01 (**E**) Gene expression levels of *Sall4*, *Piwil 1*, *Nanos2*, and *Dazl* from each experiment group. The graph shows mean ± SD with log 2 scale. *n* = 5, ** *p* < 0.01. (**F**) Hematoxylin and eosin staining of testis from each group, showing immunostaining of SALL4 in testes. Scale bar = 100 μm. (**G**) The number of DDX4+ and SALL4+ cells from seminiferous tubules. At least 50 tubules were scored for each testis (five to seven biological replicates). The graph is shown as mean ± SD, and significance levels between Ctrl and treated are presented as asterisks *** *p* < 0.001. (**H**) Protein expression levels of SALL4 in testis from each group. The graph shows relative protein expressions normalized to β Actin (*n* = 3). ** *p* < 0.01.

The testicular diameter decreased after administering PRO to mice for eight weeks, although it did not affect the body weight of the mice or the weight of the testes (Figure 1A–C). The diameter of seminiferous tubules in PRO groups appeared to be smaller than that of the Ctrl group (Figure 1D). The gene expression of early-stage germ cells before meiosis stage markers such as *Sall4*, *Piwil*, *Nanos2*, and *Dazl* was evaluated, and the results showed that all genes were significantly downregulated by the PRO treatment (Figure 1E). A histological analysis of the testes from each group was performed. Similar to the diameter measurements, the size of the tubules appeared to be smaller than that of the Ctrl group. In addition, immunostaining with Sall4 antibody demonstrated that Sall4+ cells were more abundant in the testes of the Ctrl group than in the PRO-treated group (Figure 1F). Quantitative analysis of Sall4+ cells revealed a significant decrease in the testes in the PRO-treated group (Figure 1G). The protein expression of Sall4 was investigated in each group to verify the reduction in Sall4+ cells in the PRO-treated groups. Consistently, Sall4 protein was expressed at higher levels in the Ctrl group than in the RPO-treated group (Figure 1H).

### 3.2. Effects of PRO Steroidogenesis and Sperm Motility in Mice Testis

**Figure 2:**
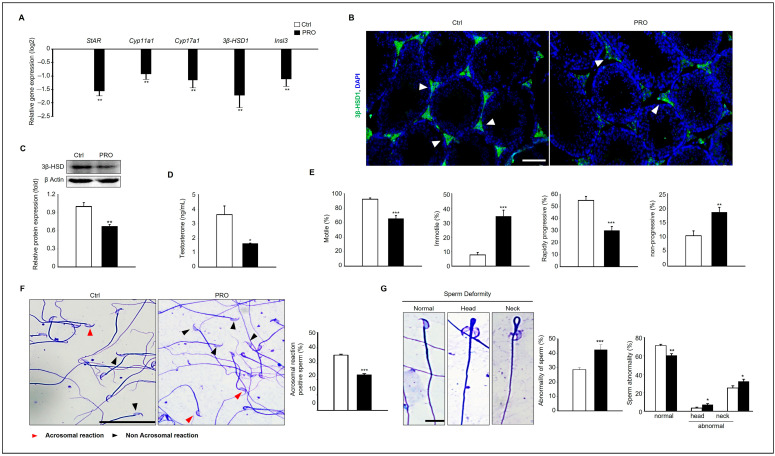
PRO exposure affects Sertoli cells and steroidogenesis in the testes. (**A**) Gene expression levels of StAR, Cyp11a1, Cyp17a1, 3β-HSD1, and Insl3 in the testis from each group. The graph represents mean ± SD with log2 scale (*n* = 3). ** *p* < 0.01. (**B**) Immunostaining of 3β-HSD1 in the testes of PRO- treated and Ctrl mice (arrows). Scale bar = 100 µm. (**C**) The protein expression of 3β-HSD1 from each group. The graph shows relative protein expressions normalized to β actin (*n* = 3). ** *p* < 0.01. (**D**) Concentration of serum testosterone in PRO-treated and Ctrl mice. The graph represents mean ± SD (*n* = 5). * *p* < 0.05. (**E**) Comparison of sperm motility and parameters including motile (%), immotile (%), Rapidly progressive (%), and non-progressive (%). At least 500 sperm were analyzed. The graph presents mean ±  SD. ** *p*  <  0.01, *** *p*  <  0.001. (**F**) Coomassie-stained sperm cells of PRO-treated and Ctrl mice. Red arrowheads indicate that an acrosomal reaction took place, and black arrowheads indicate that an acrosomal reaction did not take place. The graph represents the percentage of acrosomal reaction-positive sperm (%) as mean ± SD (analyzed 1000~1500 sperm in each group). Scale bar = 50 μm. (**G**) The image of sperm shows normal sperm in Ctrl and a prominent deformity in the sperm head and neck. The tail was normal in all groups. 1000~1500 sperm were analyzed in each group. The graph shows the percentage of abnormality of sperm (%). Scale bar = 10 μm. Data are expressed as mean  ±  SD. * *p*  <  0.05, ** *p*  <  0.01, *** *p*  <  0.001.

We previously found that PRO has negative effects on germ cells in the testes. We also evaluated whether PRO affected steroidogenesis in the testes. As a result, the expression of genes related to steroidogenesis such as *StAR*, *Cyp11a1*, *Cyp17a1*, and *3-βHSD1* was significantly downregulated by the PRO treatment. The gene expression of *Insl3,* which is known as a Leydig cell marker, was also downregulated by RPO (Figure 2A). To verify these results, the protein expression of 3β-HSD1 was detected by immunostaining and Western blotting analysis. The result showed that 3β-HSD1 was rarely observed in the intertidal area of PRO-treated groups compared to the Ctrl (Figure 2B). Consistently, 3β-HSD1 protein was also more abundant and detected in the Ctrl group than the PRO-treated group (Figure 2C).

Serum testosterone levels in the PRO-treated group (1.63 ng/mL) were significantly decreased compared to that in the Ctrl group (3.63 ng/mL) (Figure 2D). There is evidence that sperm parameters decrease because of abnormal steroidogenesis [21,22]. Velocity straight line (VSL), velocity curvilinear (VCL), velocity average path (VAP), linearity (LIN), straightness (STR), wobble (WOB), motility (%), immotility (%), rapid progression (%), and non-progression (%) were evaluated. The results demonstrated that VSL/s, LIN (%), STR (%), motility (%), and rapid progression (%) were significantly reduced in the RPO-treated group compared with the Ctrl. In contrast, immotility (%) and non-progression (%) were increased in the PRO-treated group (Figure 2E and Supplementary Data). The data from Coomassie-stained sperm cells of PRO-treated and Ctrl mice reveals that acrosomal reaction significantly decreased in the sperm of the PRO-treated groups compared to Ctrl (Figure 2F). In addition, deformities in sperm head and neck were analyzed in each experimental group. The percentage of sperm abnormalities was higher in the PRO-treated group than in the Ctrl group. Notably, prominent deformities in the sperm head and neck were observed in the PRO-treated group compared to the Ctrl. The tail appeared in the normal groups (Figure 2G).

### 3.3. PRO Induces Germ Cell Damage via Autophagy and Pro-Apoptosis in the Testis

**Figure 3:**
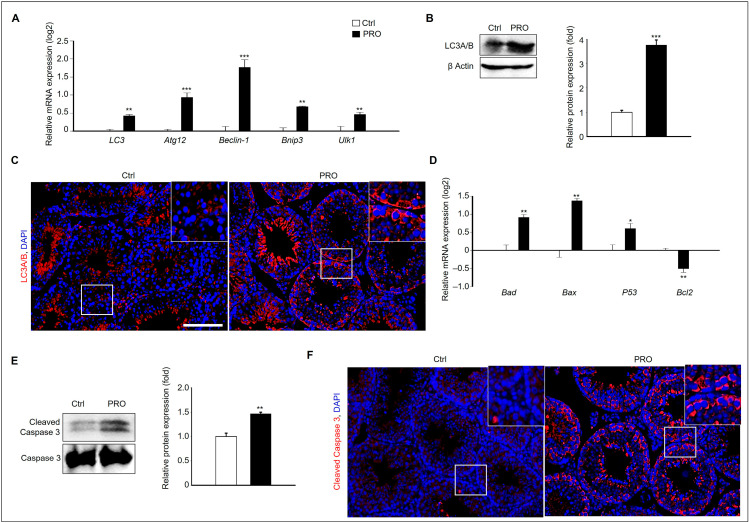
The pro-apoptotic effect via autophagy of RPO in testis germ cells. (**A**) Gene expression of *LC3*, *Atg12*, *Beclin-1*, *Bnip3*, and *Ulk1* in the testis from each group. The graph shows mean ± SD with log2 scale (*n* = 3). ** *p* < 0.01, *** *p* < 0.001. (**B**) Expression of LC3A/B protein in the testis of each group. The graph shows relative protein expressions normalized to β Actin (*n* = 3). *** *p* < 0.001. (**C**) Immunostaining of LC3A/B in the testes of PRO-treated and Ctrl mice (*n* = 3). Scale bar = 100 µm. (**D**) Gene expression of *Bad*, *Bax*, *Caspase3*, and *P53* in the testis from each group. The graph shows mean ± SD with log2 scale (*n* = 3). * *p* < 0.05 and ** *p* < 0.01. (**E**) Expression of Cleaved Caspase3 protein in the testis of each group. The graph shows relative protein expressions normalized to Caspase 3. ** *p* < 0.01. (**F**) Immunostaining of Cleaved Caspase3 in the testes of PRO-treated and Ctrl mice (*n* = 3). Scale bar = 100 µm. Locate of germ cell which near the basement membrane (white box).

Before conducting mechanistic studies using GC-1 spg cells which is spermatogonia, we confirmed the expression of autophagy-related genes and proteins to investigate the potential mechanisms underlying PRO-induced damage to testicular germ cells in testes. In PRO-treated testes, the expression of autophagy-related genes, including *LC3*, *Atg12*, *Beclin-1*, *Bnip3*, and *Ulk1*, as well as LC3A/B proteins, was significantly increased compared to the Ctrl group (Figure 3A,B). To further localize autophagy activation, immunohistochemical staining was performed using an LC3A/B antibody, which revealed enhanced LC3A/B expression specifically in germ cells attached to the basement membrane following PRO exposure (Figure 3C). Additionally, PRO treatment upregulated the expression of pro-apoptotic genes such as *Bad*, *Bax*, *Caspase-3*, and *P53*, indicating the induction of apoptosis in testicular tissue (Figure 3D). Protein analysis showed a marked increase in cleaved caspase-3 expression (Figure 3E), and immunostaining confirmed that this increase was localized specifically to germ cells in PRO exposed testes (Figure 3F). Taken together, these results suggest that PRO promotes germ cell apoptosis in the testes, potentially through activation of the autophagy pathway.

### 3.4. PRO Induces GC-1 Spermatogonia Cellular Damage via a Pro-Apoptotic Pathway

**Figure 4:**
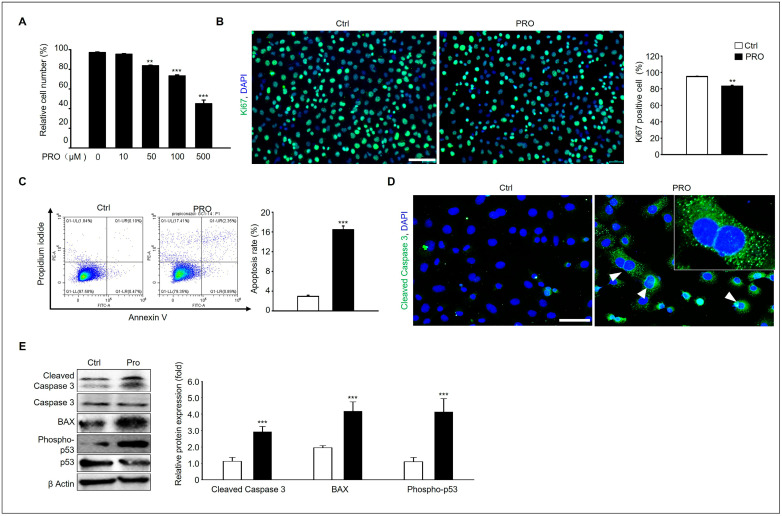
The pro-apoptotic effect of RPO in GC-1 spg cells. (**A**) The viability of GC-1 spg cells was assessed using an MTT assay, after a 24 h treatment with 0–500 μM PRO. The differences in viability between the PRO-treated groups and the 0 μM Ctrl group were statistically significant. All data are expressed as the mean ± SD from three independent experiments (*n* = 5, ** *p* < 0.01, *** *p* < 0.001). (**B**) Immunofluorescence analysis of Ki-67 in GC-1 spg cells after culture with or without 100 μM PRO. Scale bar = 100 μm. Quantification of Ki-67-positive cells (green) with respect to total cells (blue) (%). Data are presented as mean ± SD (*n* = 4, ** *p* < 0.01). (**C**) The apoptotic cell death of GC-1 spg cells due to 100 μM PRO exposure was evaluated using flow cytometry. Apoptosis was identified through Annexin V-FITC/PI staining. The graph illustrates the apoptosis rates, with all data presented as mean ± SD from three independent experiments. Significance levels between the Ctrl and treated groups are indicated by asterisks (*n* = 5, *** *p* < 0.001). (**D**) Immunostaining of cleaved caspase 3 in each sample (white arrowhead), and DAPI-stained nucleus (*n* = 4). Scale bar = 50 μm. (**E**) GC-1 spg cells were treated with or without 100 μM PRO for 24 h, and the total protein was prepared and analyzed by Western blotting. The protein expression levels of cleaved caspases 3, caspase 3, phospho-p53, p53, BAX, and β Actin in each experimental group. The graph represents mean ± SD, and relative protein expression is normalized to an inactive form or β Actin (*n* = 3). *** *p* < 0.001.

The toxic effects and mechanisms of PRO have therefore been studied in detail in GC-1 spermatogonial (spg) cells. The cell viability assays were conducted on GC-1 spg cells following RPO exposure (0–500 μM) to assess the cytotoxicity of PRO. The results demonstrated a dose-dependent decrease in cell viability when the cells were exposed to 50–300 µM PRO for 24 h, compared to the Ctrl group (0 µM PRO). Based on these findings, the LC50 value was determined to be 54.53 μM (Figure 4A) and we selected concentrations of 100 μM PRO for this study, and this dosage also reflects the previous study [3,5]. To investigate the effect of PRO on cell proliferation, immunostaining was performed using a Ki-67 antibody (Figure 4B). The number of Ki-67-positive cells (indicated by FITC positivity) declined in the GC-1 spg cells exposed to 100 μM PRO. Figure 3B illustrates the percentage of Ki-67-positive cells relative to the total cell count (%) during the active phase of the cell cycle, revealing a significant reduction in Ki-67-positive cells when treated with 100 μM PRO. Flow cytometry was performed using the Dead Cell Apoptosis Kit to determine the proportion of apoptotic cells (both in the early and late stages) among GC-1 spg cells exposed to PRO. As depicted in Figure 3E, there was a notable increase in the apoptosis rate in cells 24 h after exposure to 100 μM PRO compared to untreated Ctrl. More than 16% of the cells were apoptotic in the groups exposed to 100 μM PRO (Figure 4C). Based on the flow cytometry results, the expression levels of pro-apoptotic proteins were assessed in GC-1 cells exposed to PRO. Immunostaining indicated the presence of cleaved caspase 3 in the PRO-exposed samples, while it was absent in the Ctrl group (Figure 4D). To confirm these findings, the protein expression levels of cleaved caspase 3, caspase 3, BAX, phospho-p53, p53, and beta-actin were measured in each sample. The results indicated that the protein expression of active forms such as Cleaved caspase 3, Phospho-p53, and BAX was significantly increased in PRO-treated samples compared to that in the Ctrl (Figure 4E).

### 3.5. PRO Induces Autophagic Cell Death in GC-1 Spg Cells

**Figure 5:**
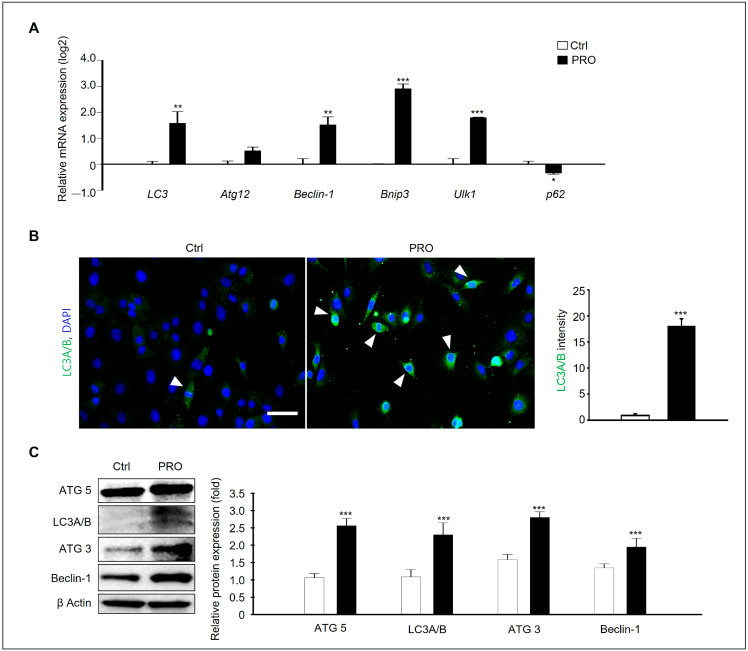
PRO-induced GC-1 spg cell damage mediated via autophagy. (**A**) qPCR was used to assess the expression of autophagy-related genes, including LC3, Atg12, Beclin-1, Bnip3, UlK1, and p62 in cells exposed to 0 or 100 μM PRO for 24 h. Data are presented as mean ± SD, with log2 values. * *p* < 0.05, ** *p* < 0.01, and *** *p* < 0.001 indicating significant differences compared to the Ctrl (*n* = 3). (**B**) Immunofluorescence analysis showed LC3A/B expression in GC-1 spg cells treated with 0 or 100 μM PRO (white arrowheads), with cell nuclei stained using DAPI. Scale bar= 50 μm. The graph shows the intensity of LC3A/B using Image J software (version 1.54; NIH image, Bethesda, MD, USA). Data represents the mean ± SD (*n* = 5, *** *p* < 0.001 compared to the Ctrl). (**C**) Western blot analysis was performed to measure the levels of ATG5, LC3A/B, ATG3, and Beclin-1 in PRO-treated GC-1 spg cells, with densitometric analysis normalized to β Actin. Values represent the mean ± SD from three independent experiments (*n* = 3, *** *p* < 0.001 compared to the Ctrl).

Next, we investigated the mechanism of PRO-induced damage to GC-1 spg cells. Generally, cellular damage and apoptosis are related to autophagy, reactive oxygen species (ROS), and endoplasmic reticulum (ER) stress. We investigated whether PRO triggers autophagy in GC-1 spg cells despite the known induction of apoptotic cell death. Our findings revealed that the expression levels of autophagy-related genes, including LC3, ATG12, Beclin1, Bnip3, and Ulk1, were significantly elevated in the GC-1 spg cells treated with 100 μM PRO compared to the Ctrl. P62, also known as SQSTM1 (sequestosome 1), is a factor degraded by autophagy, and PRO decreased the genetic expression level of *p62* in the GC-1 spg cells (Figure 5A). Immunostaining for LC3A/B demonstrated a notable increase in LC3A/B-positive cells in the PRO-treated groups, as observed by confocal microscopy. The intensity of LC3A/B was also significantly higher in PRO-treated samples than in Ctrl (Figure 5B). Western blot analysis presented consistently higher levels of Atg5, LC3A/B, ATG3, and Beclin-1 proteins in PRO-treated cells than in the Ctrl (Figure 5C). These results indicated that PRO induces autophagy and apoptotic cell death in GC-1 spg cells.

### 3.6. 3-Methyladenine (3-MA) Protects Against PRO-Induced Autophagy in GC-1 Spg Cells

**Figure 6:**
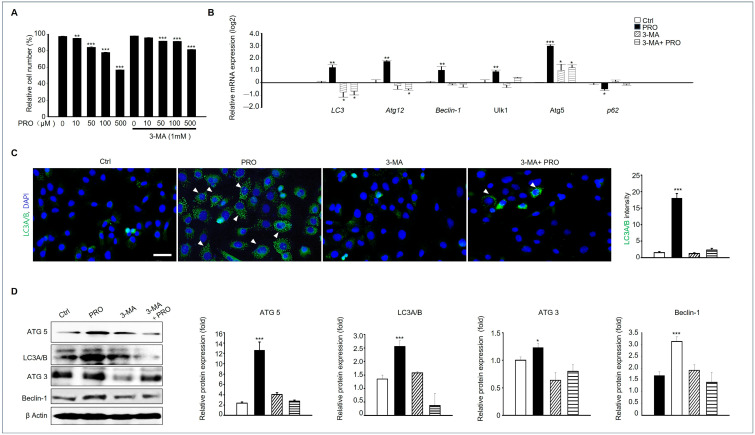
3-MA prevents autophagy in GC-1 spg by PRO treatment. (**A**) The viability of GC-1 spg cells was assessed using the MTT assay after 24 h of treatment with 0–500 μM PRO with or without 3-MA (1 mM). ** *p* < 0.01, *** *p* < 0.001. (**B**) Gene expression levels of *LC3*, *Atg12*, *Beclin-1*, *Ulk1*, *Atg5* and *p62* in each group, as determined by qPCR. Data are represented as mean ± SD (*n* = 5). * *p* < 0.05, ** *p* < 0.01, *** *p* < 0.001 compared to Ctrl. (**C**) Immunostaining of LC3A/B (white arrowheads) from each group (Ctrl, PRO, 3-MA, PRO + 3-MA). Nuclei were stained. Scaler bar = 50 μm. The graph represents LC3A/B intensity with mean ± SD (*n* = 5). *** *p* < 0.01 compared to Ctrl. (**D**) Protein expression of ATG5, LC3A/B, ATG3, and Beclin-1 in each group. Each protein expression is relative to β Actin, which is the loading Ctrl shown as a graph with fold values and mean ± SD (*n* = 3). * *p* < 0.05 and *** *p* < 0.001 compared to Ctrl.

Based on our findings presented in Figure 5, autophagy is involved in GC-1 spg cellular damage caused by PRO. To clarify this, we investigated whether 3-MA, an autophagy inhibitor, prevented PRO-induced autophagy. In Figure 6A, 3-MA at 1 mM enhanced the cell viability of GC-1spg treated with Pro (0–500 μM). The expression of autophagy markers such as LC3, Atg12, beclin-1, Ulk1 and Atg5 was investigated, and the results revealed that the expression of all genes was significantly increased in PRO-treated groups, but not in the 3-MA-treated groups with PRO. On the other hand, the expression of p62 was decreased in the PRO-treated group, but 3-MA did not show a significant difference compared with the Ctrl group (Figure 6B). Immunostaining results demonstrated that the LC3A/B signal was abundant in the PRO-treated groups, but not in the RPO-treated with 3-MA groups. The intensity of LC3A/B was also higher in the PRO-exposed GC-1 spg cells than in the other groups (Figure 6C). To confirm these findings, we examined the protein expression of autophagy markers ATG5, LC3A/B, ATG3, and Beclin-1. The results revealed that the expression of all these proteins was significantly increased in the groups treated with PRO alone, but not in the groups treated with 3-MA and PRO (Figure 6D).

### 3.7. Inhibition of Autophagy Can Suppress the Pro-Apoptotic Pathway of GC-1 Spg by PRO

**Figure 7:**
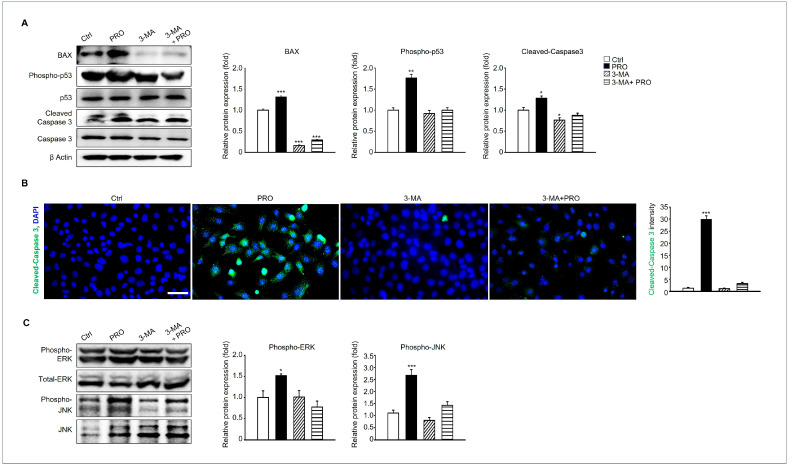
The effects of 3-MA on PRO-induced apoptosis. (**A**) Protein expression of BAX, Phospho-p53, p53, Cleaved caspase 3, Caspase 3, and β Actin in each experimental group. Data means SD (*n* = 3). BAX protein normalized to β Actin levels, phospho-p53, and cleaved caspase 3 were normalized to an inactive form. * *p* < 0.05, ** *p* < 0.01 and *** *p* < 0.001. (**B**) Immunostaining of cleaved caspase 3 in each experimental group. Scale bar= 50 μm. The graph shows the intensity of cleaved-caspase 3 using Image J software. Data represents the mean ± SD (*n* = 4, *** *p* < 0.001 compared to the Ctrl). (**C**) Protein expressions of phopsho-ERK, total ERK, phospho-JNK, and total-JNK were detected in each experimental group. Protein expressions of phospho-ERK and phospho-JNK were normalized to total-ERK and JNK. The graph shows the mean ± SD (*n* = 3, * *p* < 0.05, *** *p* < 0.001 compared to Ctrl).

Our previous results demonstrated that the expression of pro-apoptotic protein significantly increased in the PRO-treated group (Figure 4E). We, therefore, investigated whether the expression of apoptotic proteins was regulated by the autophagy inhibitor 3-MA. The expression levels of pro-apoptotic proteins such as BAX, phosphorylated p53, and cleaved caspase 3 were increased in the PRO-treated groups, but not in the PRO + 3-MA-treated groups (Figure 7A). Consistently, immunostaining of cleaved-caspase 3 also showed that strong signals were detected in the PRO groups but not in either the 3-MA- or PRO-treated groups (Figure 7B). MAPK (mitogen-activated protein kinase) signaling pathways control numerous biological processes through various cellular mechanisms such as apoptosis, and autophagy [23]. Based on these references, the expression of phosphorylated-ERK1/2 and JNK, which are major molecules involved in MAPK signaling, was detected in each group. The results showed that phosphorylated-ERK1/2 and JNK were highly detected in the PRO-treated groups but not in the other groups. In contrast, MAPK signaling was increased by PRO and suppressed by an autophagy inhibitor (Figure 7C).

## 4. Discussion

Triazoles are common fungicides that are widely used in agriculture, easily found in the natural environment, and detectable in organisms. However, their effects on living and developing animals remain largely unknown. Our study focused on the effects of PRO on male testes and elucidated the molecular mechanisms underlying PRO toxicity in testicular cells. The current study is significant because it is the first study on the male reproductive function and toxicity of PRO in mammals, especially rodents.

Studies of PRO toxicity in mice are rare. Several studies have shown that PRO enhances the development of hepatic preneoplastic foci in rats following treatment with dimethyl nitrosamine. In addition, PRO causes hepatomegaly-induced hepatic necrosis in rats at a dose of 450 mg/kg [4,24]. Murphy et al. further reported that PRO promotes liver cell proliferation by disrupting the cholesterol biosynthesis pathway, which in turn activates Erk1/2 through farnesylation of Ras [25].

Several studies have reported the effects of azoles on reproductive function and organs. In male mice, tebuconazole exposure led to a decrease in germ cell number in the fetal testes [26], and fipronil affected proteins related to sperm function, which affected male fertility [27]. In female mice, prothioconazole (PTC) exposure has the potential to harm oocyte development and fertilization by causing mitochondrial dysfunction, oxidative stress, and apoptosis [28]. In zebrafish, exposure to difenoconazole (DCZ) reduced egg production, fertilization rates, and gamete frequency at higher concentrations (10 μg/L). It also disrupted hormone levels such as 17β-estradiol (E2), testosterone (T), and vitellogenin (VTG) and significantly reduced male–female contact time as reproductive behavior [29].

Regarding male reproduction, there has been one report of toxicity following azole exposure, although not in rodents. Svanholm et al. reported that azole fungicides disrupted spermatogenesis and related hormonal effects in peripubertal *X. tropicalis*. Elevated levels of testicular aldh1a2 were found in male rats exposed to PRO, although no histological changes were noted. In another common azole, imazalil (IMZ), histological observations of the testes indicated a higher number of dark spermatogonial stem cells (SSCs) per unit area and an increased ratio of secondary spermatogonia to dark SSCs in IMZ-treated groups relative to the ctrl. Testicular levels of 3 β-HSD and DDX4 were elevated, whereas cyp19 and ID4 levels were lower in the IMZ-treated groups [30]. In our study, the cellular marker of early-stage germ cells before the meiotic division was significantly decreased in the testes of RPO-treated mice compared to that in smaller than that of the ctrl group. Based on these results, we investigated the molecular mechanisms underlying PRO toxicity in spermatogonia, which GC-1 spg.

Several in vitro studies have demonstrated that PRO affects various aspects of steroidogenesis and the functions of specific hormone receptors, indicating its potential as an endocrine disruptor. In addition, PRO exerts an antagonistic effect on androgen receptors [10]. This previous report supports our results, presenting a reduction in steroidogenesis in the testes following PRO exposure in mice. In our in vivo results, steroidogenic markers including StAR, Cyp11a1, Cyp17a1, 3β HSD1, and Insl3 were reduced by the PRO treatment in testis. Sperm parameters were additionally negatively regulated by PRO. This result suggests that a decrease in steroidogenesis may have contributed to the observed decrease in sperm activity such as various parameters.

One report has described the effect of PRO on the development of *X. tropicalis*. PRO induced endocrine changes during a key phase of sexual development in *X. tropicalis*, resulting in ongoing reproductive and liver damage visible two months after treatment ended. During metamorphosis, brain aromatase activity was markedly elevated in the high-dose PRO group compared to the ctrl group. The PRO-treated groups had a higher rate of individuals reaching metamorphosis sooner than the ctrl group. Additionally, two months after metamorphosis, males in the low-dose groups exhibited smaller testis size, reduced sperm, and Müllerian duct maturity [6].

Several research groups have previously investigated triazoles. In EA.hy 926 endothelial cells, ipconazole exposure induces cellular cytotoxicity via ROS and pro-inflammatory responses [31]. Among of Triazole series, several studies have reported on the cellular toxicity mechanism of PRO. Nesnow et al. reported that PRO increased ROS levels in mouse hepatic AML12 cells. PRO treatment also elevated glutathione-S-transferase (GST) protein levels and thiobarbituric acid-reactive substances (TBARS) in AML12 cells. TBARS levels were reduced by diphenylene iodonium chloride (DPIC), a cytochrome P450 (CYP) reductase inhibitor, indicating that CYPs play a role in ROS generation [32].

Our results demonstrated that PRO induced apoptosis in GC-1 spg cells via autophagy signaling. Although there are no reports on autophagy-mediated cellular damage or death caused by PRO, autophagy-mediated cellular damage caused by several other types of triazoles has been reported. For example, cyclobutanil causes neurotoxicity in zebrafish larvae (*Danio rerio*) by triggering both autophagy and apoptosis via the enhancement of LC3-LC3- II protein and suppression of p62 protein expression [33]. Difenoconazole is also one of the triazole fungicides. Exposure to difenoconazole led to cardiotoxicity in carp (*Cyprinus carpio*), mediated by oxidative stress, inflammation, apoptosis, and autophagy [34]. Itraconazole also induces autophagic progression in human glioblastoma cells [35].

Autophagy is a conserved lysosome-dependent process essential for the degradation and recycling of cellular components. Emerging evidence links autophagy to male reproductive health by influencing spermatogenesis and endocrine function. Problems such as a declining sperm count, erectile dysfunction, and infertility have intensified in recent decades, affecting the overall health of aging men [36].

There are insufficient reports on the effects of PRO on male reproduction. This study is the first to analyze the effects of PRO on germ cells in detail.

Based on our in vivo study, we described the cellular toxicity of PRO on GC-1 spg cells, which are type B spermatogonia and are known as germ cells in the testes. The study of spermatogonial toxicity is critical because spermatogonia forms the foundation of male fertility. These cells give rise to sperms, which undergo several stages of differentiation and division, eventually becoming mature spermatozoa. Any damage or toxicity to these cells can impair this process, leading to reduced sperm production and potential infertility. Germ cells also interact with Sertoli cells and other components of the testicular environment, influencing hormonal regulation and the overall health of the male reproductive system. Damage to germ cells can disrupt these interactions, thereby affecting hormonal balance and reproductive health [37,38].

Cellular stressors such as oxidative damage, DNA lesions, and inflammatory cytokines can stimulate the MAPK signaling network, including JNK and p38 pathways, which subsequently activate apoptotic signaling through downstream effectors. In contrast, ERK is mainly involved in promoting cell survival and proliferation; however, depending on the context, either its suppression or aberrant activation may also trigger apoptosis. Thus, MAPK cascades serve as critical mediators that translate environmental stress signals into programmed cell death, maintaining cellular homeostasis under harmful conditions. In addition, autophagy could induce apoptosis in response to environmental stress [39]. In our study, treatment with an autophagy inhibitor suppressed the expression of molecules related to apoptosis and MAPK signaling, which is consistent with the findings described above.

Our results showed that germ cells were damaged by PRO exposure, both in vivo and in vitro. In particular, the detailed mechanism underlying intracellular PRO toxicity was identified in GC-1 cells. The impact of pesticides on the male reproductive system includes various adverse effects, such as reduced sperm quality and quantity, hormonal imbalances, and potential damage to reproductive organs. These effects can result in reduced fertility and other reproductive health problems [40]. PRO has been found in human hair samples from the general population, suggesting environmental exposure [8,41]. These facts and our findings suggest that PRO may have negative effects on reproductive function, particularly in humans. Although the in vivo experiment in this study confirmed the reproductive toxicity of PRO at a concentration of 150 mg/kg, which is relatively high compared to the levels detected in human hair, the in vitro experiment elucidated the precise mechanism of PRO-induced germ cell toxicity. The exposure dose of PRO depends on environmental conditions, exposure duration, and frequency. Further studies are needed to investigate the effects of various concentrations (both high and low) of PRO exposure on in vivo reproductive toxicity.

## 5. Conclusions

In summary, the current study investigates the toxicity effects of PRO on the male reproductive system in mice using an in vivo model. Additionally, the detailed molecular mechanism of PRO toxicity on germs was examined. In vivo, the result demonstrated that undifferentiated germs were damaged by PRO, and reduced steroidogenesis and sperm parameters were observed in the testes following PRO exposure. In germ cells, apoptosis and autophagy markers were upregulated following PRO treatment of GC-1 cells. In addition, the upregulated expression of apoptosis-related markers in RPO-exposed cells was reduced by 3-MA treatment. This study is the first to demonstrate damage to the male reproductive system in mice by PRO and its underlying mechanism.

## Figures and Tables

**Table 1:** List of antibodies used for immunostaining (Western blotting).

Antibody	Company	Catalog Number	Dilution, IHC (WB)
DDX4	Abcam	ab13840	1:300
SALL4	Santa Cruz Biotech	SC-101147	1:200 (1:1000)
3β-HSD	Santa Cruz Biotech	SC-30820	1:200 (1:1000)
β Actin	Santa Cruz Biotech	SC-47778	1:200 (1:1000)
Ki67	Abcam	ab15580	1:300
Cleaved Caspase 3	Cell signaling	#12994	1:300 (1:1000)
Caspase 3	Cell signaling	#9662	(1:1000)
LC3A/B	Cell signaling	#12741	1:300 (1:1000)
ATG5	Cell signaling	#12994	(1:1000)
ATG3	Cell signaling	#3415	(1:1000)
Beclin-1	Cell signaling	#3738	(1:1000)
phospho-Erk1/2	Cell signaling	#4370	(1:1000)
Total-ERK1/2	Cell signaling	#9102	(1:1000)
phospho-JNK	Cell signaling	#4668	(1:1000)
JNK	Cell signaling	#9252	(1:1000)
BAX	Cell signaling	#2772	(1:1000)
phospho-p53	Cell signaling	#3556	(1:1000)
p53	Cell signaling	#2524	(1:1000)

**Table 2:** Primers used for reverse transcription-polymerase chain reaction using mouse cDNA.

Gene	Forward Primer	Reverse Primer
*Gapdh*	5′-GTCGGTGTGAACGGATTTG-3′	5′-CTTGCCGTGGGTAGAGTCAT-3′
*Sall4*	5′-TCTCAGCAAGTGTCCGTGTC-3′	5′-GCATGAGGTAGCTTGGCTTG-3
*Piwil 1*	5′-TGGTGATTGGAATGGATGTG-3	5′-TGGTGATTGGAATGGATGTG-3
*Nanos2*	5′-ATAATTCAGAGCCGGAAGCA-3′	5′-TCTTCAGCTGGTGTGAGGTG-3′
*DAZL*	5′-GTCGAAGGGCTATGGATTTG-3′	5′-ACGTGGCTGCACATGATAAG-3′
*StAR*	5′-TGGGCATACTCAACAACCAG-3	5′-GTCTACCACCACCTCCAAGC-3
*Cyp11a1*	5′-GACAATGGTTGGCTAAACCTG-3	5′-GGGTCCACGATGTAAACTGAC-3
*Cyp17a1*	5′-TCCAGCATTGGAGAGTTTGC-3	5′-ATGAGATGGCTTCCTGTTGG-3
*3β-HSD1*	5′-AATCTGAAAGGTACCCAGAA-3′	5′-TCATCATAGCTTTGGTGAGG-3
*Insl3*	5′-TGCAGTGGCTAGAGCAGAGAC-3′	5′-GAGAAGCCTGGAGAGGAAGC-3
*Ulk1*	5′-ACACACCTTCTCCCCAAGTG-3	5′-GACGCACAACATGGAAGTCG-3
*Bnip3*	5′-GCTCCTGGGTAGAACTGCAC-3′	5′-GCTGGGCATCCAACAGTATT-3′
*Beclin-1*	5′-GCGGGAGTATAGTGAGTT-3ʹ	5′-GGTGGCATTGAAGACATT-3
*Atg12*	5′-TAAACTGGTGGCCTCGGAAC-3	5′-ATCCCCATGCCTGGGATTTG-3′
*Atg5*	5′-ACTTGCTTTACTCTCTATCAG-3	5′-CATCTTCTTGTCTCATAACCT-3
*LC3*	5′-CTTCGCCGACCGCTGTAA-3′	5′-GCCGGATGATCTTGACCAACT-3′

## Data Availability

Data will be made available on request.

## Supplementary Materials

The following supporting information can be downloaded at: https://www.mdpi.com/article/10.3390/cells14201624/s1. Supplmentary Data S1: Comparison of sperm motility and parameters including Velocity Straight Line (VSL), Velocity Curvilinear (VCL), Velocity Average Path (VAP), Linearity (LIN), Straightness (STR), Wobble (WOB). (analyzed at leat 1500 sperm). Graph presented mean ± SD. ** *p* < 0.01, *** *p* < 0.001.
