# Supplementary material for: Propiconazole-Induced Testis Damage and MAPK-Mediated Apoptosis and Autophagy in Germ Cells
Source: Cells. 2025 Oct 17;14(20):1624. doi: 10.3390/cells14201624 (PMC12564799; doi:10.3390/cells14201624)

Supplmentary Data S1. Comparison of sperm motility and parameters including Velocity Straight Line (VSL), Velocity Curvilinear (VCL), Velocity Average Path (VAP), Linearity (LIN), Straightness (STR), Wobble (WOB). (analyzed at leat 1500 sperm). Graph presented mean± SD. $**P < 0.01$, $***P < 0.001$.

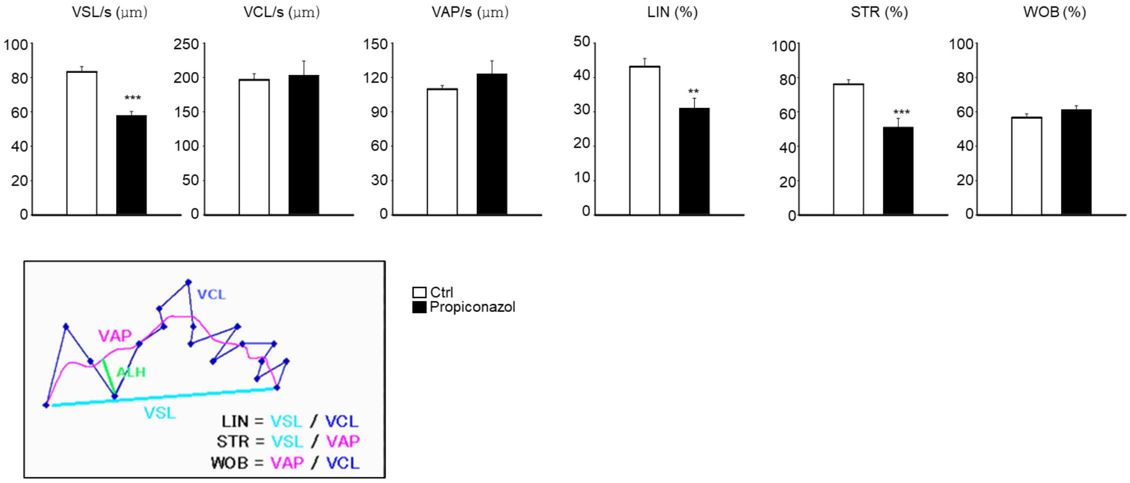

Supplement: Supplementary file 1 [file cells-14-01624-s001.zip › cells-3909262-supplementary.pdf]
